# Trends in age-standardised prevalence of type 2 diabetes mellitus according to country from 1990 to 2017 and their association with socioeconomic, lifestyle and health indicators: An ecological study

**DOI:** 10.7189/jogh.11.04005

**Published:** 2021-01-31

**Authors:** Yoshiro Shirai, Tomoko Imai, Ayako Sezaki, Keiko Miyamoto, Fumiya Kawase, Chisato Abe, Masayo Sanada, Ayaka Inden, Takumi Kato, Norie Suzuki, Hiroshi Shimokata

**Affiliations:** 1 Department of Food and Nutritional Environment, Kinjo Gakuin University, Aichi, Japan; 2 Department of Food Science and Nutrition, Doshisha Women’s College of Liberal Arts, Kyoto, Japan; 3 Graduate School of Nutritional Sciences, Nagoya University of Arts and Sciences, Aichi, Japan; 4 Department of Food Science and Human Nutrition, Ryukoku University, Shiga, Japan; 5 Department of Nursing, Nagoya University of Arts and Sciences, Aichi, Japan; 6 Department of Nutrition, Asuke Hospital Aichi Prefectural Welfare Federation of Agricultural Cooperatives, Aichi, Japan; 7 Department of Life and Environmental Science, Tsu City College, Mie, Japan; 8 Hamamatsu University Hospital, Shizuoka, Japan; 9 Japanese Red Cross Nagoya Daini Hospital, Aichi, Japan; 10 Faculty of Core Research, Ochanomizu University, Tokyo, Japan

## Abstract

**Background:**

There is a need for global public health strategies to effectively curb the ever-growing global diabetes population. This longitudinal ecological study was conducted to elucidate the country-specific trends of the age-standardised prevalence of type 2 diabetes mellitus (T2DM) and their association with socioeconomic, lifestyle and health indicators.

**Methods:**

Data pertaining to the age-standardised prevalence of T2DM (rates per 100 000) and socioeconomic, lifestyle and health indicators were obtained from several international databases. Data available from 1990 to 2017 for 139 countries with populations of 1 million or greater were analysed, followed by estimation of slopes for T2DM prevalence in each country. The longitudinal association between T2DM and the standardised variables of socioeconomic, lifestyle and health indicators was fitted using a generalised linear mixed-effects model with random intercept for each country and random slope for year.

**Results:**

The country-specific age-standardised prevalence of T2DM decreased significantly in 9 countries, remained unchanged in 11 countries and increased significantly in 119 countries. The estimated standardised effect of age-standardised education for T2DM was the largest at −524.5 (SE; 109.9), followed by −514.8 (SE; 95.6) for physical activity and 398.8 (SE; 45.8) for body mass index (BMI) (*P* < 0.0001 for all).

**Conclusions:**

The factors largely associated with global T2DM prevalence and trends were years of education, followed by physical activity and BMI. This study also provides basic resources for examining public health approaches to curb the increase in global T2DM prevalence.

The increasing global prevalence of diabetes has emerged as one of the important public health issues. According to periodic diabetes prevalence reports of the International Diabetes Federation (IDF), the worldwide number of people with diabetes was 425 million in 2017 that is expected to continue to increase [1]. Previous reviews and meta-analyses suggest that type 2 diabetes mellitus (T2DM) is a preventable disease [2,3]. It is important to control the increase in the prevalence of diabetes as it presents a significant risk factor for coronary diseases [4] and contributes primarily to the causes of mortality in several countries [5]. Moreover, diabetes has a major impact on health economic costs, with approximately 12% (US$ 727 billion) of the worldwide medical expenditure being estimated to be caused due to diabetes [6]. Therefore, there exists a need for an effective global action to control the increasing worldwide diabetes prevalence. Population-level public health strategies to control the global diabetes prevalence should aim at improving the economic and educational disparities and building social support, unlike prevention measures at the individual level, as modifiable risk factors.

Currently, there are limited reports on the worldwide trends of diabetes. Ezzati et al. were the first to estimate the worldwide trends in the international comparable prevalence of diabetes, including the recent data, since 1980 [7]. Later, Vos et al. reported the worldwide prevalence of diabetes, improving the estimation method used by Ezzati et al. by not only using self-reported diabetes but also incorporating the cause of death into the model. They reported that the global age-standardised prevalence of diabetes has been increasing [8]. In addition, Murray et al. separately estimated the worldwide prevalence of type 1 diabetes and T2DM, which had not been distinguished in the past, from 1990 to 2017 [9].

A meta-analysis reported that the age-standardised prevalence of diabetes remained unchanged between 1990 and 2010 in Japan [10], amid an upward trend in the global prevalence of diabetes worldwide. The differences in trends in the age-standardised prevalence of T2DM according to country have been poorly reported, and the association between T2DM prevalence according to country and socioeconomic, lifestyle and health indicators has not been examined in a global perspective.

Therefore, we conducted this longitudinal ecological study to categorise and characterise the differences in T2DM prevalence trends according to country and to identify the socioeconomic, lifestyle and health indicators associated with T2DM at population level using international databases.

## METHODS

### Age-standardised prevalence of T2DM

To achieve the objectives of this study, we obtained data on the age-standardised T2DM prevalence rates per 100 000 population from 1990 to 2017 from the Global Burden of Diseases, Injuries, and Risk Factors Study (GBD) 2017. Data from the GBD constitute a useful and comprehensive source of comparable summary population health measures as they include country-level statistics, quantify the uncertainty and maximise the possible comparability across geographic, temporal and different health-related conditions. The GBD2017 analysis adheres to the Guidelines for Accurate and Transparent Reporting of Health Estimates (GATHER) standards developed by the WHO and other organisations. The prevalence of T2DM was estimated from the available data (including published literature, surveillance data, survey data, hospital and clinical data, and other types of data), and subsequently, data on the severity and occurrence of particular consequences of diseases, or sequelae were used to identify the proportion of prevalent cases who experienced the sequelae. Details have been reported elsewhere [9].

### Variables of socioeconomic, lifestyle and health indicators

We obtained the available data on socioeconomic, lifestyle and health indicators associated with T2DM. The age-standardised education (age standardised level of educational attainment) from 1990 to 2017 was identified from the GBD database. The percentage of population aged >65 years (ageing rate) from 1990 to 2017 and the gross domestic product (GDP) per capita (US$ 1000/capita) from 1990 to 2017 were identified from the World Bank database [11].

Food energy supply (kcal/d/capita) and alcohol supply (ethanol g/d/capita), excluding losses between production and households and reflecting consumption, were determined based on the food and agriculture data for more than 245 countries and territories from 1990 to 2017 provided by the Food and Agriculture Organization Corporate Statistical Database [12]. Age-standardised total physical activity (total physical activity (metabolic equivalent-min/week divided by 1000) for individuals above age 25, age-standardised) and smoking prevalence (age-standardised smoking prevalence, both sexes combined (proportion between 0 and 100)) from 1990 to 2017 were identified based on the GBD database.

Mean body mass index (BMI; kg/m^2^) for both men and women aged >19 years and mean systolic blood pressure (SBP; mmHg) for individuals aged >24 years from 1990 to 2017 were identified based on the GBD database.

### Statistical analysis

We analysed the country-level data available from 1990 to 2017 for 139 countries with a population of more than 1 million people. To identify trends in the prevalence of T2DM in each country and to characterise countries that apply to each level of change, the main effect of the year on T2DM according to country was assessed using a general linear model, and the 139 countries were divided into four groups according to the estimated slope as follows: G0, decrease or no change; G1, slight increase (the lowest tertile at the significant positive slope); G2, moderate increase (the middle tertile at the significant positive slope) and G3, large increase (the highest tertile at the significant positive slope). For each of the four groups, the main effects of the year on each variable of socioeconomic, lifestyle and health indicators were analysed using a generalised linear model.

Then, to explore whether the trends in those socioeconomic, lifestyle and health indicators differed among the four groups, a generalised linear mixed-effects model was used to evaluate the main effects of the year, the four groups, and their interactions on the socioeconomic, lifestyle and health indicators. The dependent and independent variables were mean-centred, and the random effects of the mixed model were the intercept for each country and the slope for year.

To investigate the factors on the socioeconomic, lifestyle and health indicators most associated with the trends of T2DM prevalence, we further analysed the longitudinal association between T2DM and socioeconomic, lifestyle and health indicators with standardised independent variables (mean 0, standard deviation 1) in each country using a generalised linear mixed-effects model by the backward stepwise selection method. The random effects of the mixed model were the intercept for each country and the slope for year. All analyses were performed using R version 3.6.3 [13], the generalised linear mixed-effects model was fitted using the ‘lme’ function of the ‘nlme’ package [14], the backward stepwise selection method was fitted using the ‘cftest’ and ‘stepAIC’ function of the ‘multcomp’ package [15] and *P* values <0.05 were considered to be statistically significant.

## RESULTS

From 1990 to 2017, the age-standardised prevalence of T2DM increased from 4576.7 to 5722.1 in the ‘Global’ of the GBD super regions (ie, in the world). The country-specific age-standardised prevalence of T2DM decreased significantly in 9 countries, remained unchanged in 11 countries (ie, 20 countries were classified as G0) and increased significantly in 119 countries. There were 37 countries (G1) below the 33^rd^ percentile (42.5) of those significant positive slope estimates, 41 countries (G2) between the 33^rd^ and 66^th^ percentiles (63.6) and 41 countries (G3) above the 66^th^ percentile (Table S1 in the **Online Supplementary Document**).

The socioeconomic, lifestyle and health indicators for the four groups in 1991, 2004 and 2017 are summarised in **Table 1**. Between 1990 and 2017, the changes in the socioeconomic, lifestyle and health indicators were largely similar among the four groups. In common among the four groups, the mean BMI changed positively (*P* < 0.05 for all), and in common with G1, G2 and G3, education and energy supply changed positively (*P* < 0.05 for all).

**Table 1:** The socioeconomic, lifestyle and health indicators for the four groups according to type 2 diabetes prevalence trends (G0 to G3)* in 1991, 2004 and 2017

	1991			2004		2017		*P*-trend†
**Mean**		**SD**	**Mean**	**SD**	**Mean**	**SD**
G0 (20 countries, in which the T2DM prevalence trends decrease or did not change)*								
Age-standardised prevalence of T2DM (per 100 000)	4952.1	1574.3		5237.9	1788.4	5231.7	1598.7	0.6465
Population (1 000 000 people)	33.4	50.2		39.0	53.0	43.5	57.5	0.6091
Aging rate (%)	8.2	4.2		9.8	5.2	11.6	6.6	0.0997
Gross domestic product (US$1000/capita)	7.9	10.0		9.1	12.7	14.7	16.8	0.1857
Age-standardised education (year)	8.0	3.8		9.3	3.6	10.4	3.4	0.0637
Mean systolic blood pressure (mmHg)	127.7	2.7		128.4	3.5	128.3	3.3	0.6050
Age-standardised smoking prevalence (%)	20.6	8.3		19.1	8.6	16.7	8.1	0.1999
Age-standardised total physical activity (MET-min/week/1000)	5.1	1.8		5.9	2.2	6.0	2.2	0.2495
Mean body mass index (kg/m^2^)	24.6	1.6		25.3	1.7	26.0	1.7	0.0167
Energy supply (kcal/d/capita)	2.6	0.5		2.8	0.5	2.9	0.4	0.0786
Alcohol supply (ethanol g/d/capita)	143.5	112.1		118.7	85.5	127.1	81.8	0.6206
G1 (39 countries, in which the T2DM prevalence trends slight increase)*								
Age-standardised prevalence of type 2 diabetes (per 100 000)	5105.7	1633.5		5524.2	1545.0	5950.5	1486.6	0.0233
Population (1 000 000 people)	28.0	30.4		29.2	36.1	33.8	44.6	0.5238
Aging rate (%)	6.8	4.5		8.9	5.5	11.3	6.5	0.0007
Gross domestic product (US$ 1000/capita)	6.1	9.4		11.2	14.6	17.0	19.0	0.0026
Age-standardised education (year)	7.3	3.1		9.0	3.2	10.2	3.0	0.0001
Mean systolic blood pressure (mmHg)	128.6	5.1		128.9	4.9	129.0	4.5	0.6898
Age-standardised smoking prevalence (%)	20.4	8.4		18.7	7.4	17.1	6.7	0.0693
Age-standardised total physical activity (MET-min/week/1000)	6.0	1.8		6.0	1.7	6.1	1.7	0.6815
Mean body mass index (kg/m^2^)	24.3	1.9		25.1	2.0	25.7	2.0	0.0028
Energy supply (kcal/d/capita)	2.7	0.6		2.9	0.5	3.0	0.5	0.0072
Alcohol supply (ethanol g/d/capita)	114.7	123.1		138.7	118.4	149.8	106.1	0.2060
G2 (39 countries, in which the T2DM prevalence trends moderate increase)*								
Age-standardised prevalence of type 2 diabetes (per 100 000)	4921.4	1422.3		6028.4	1639.7	6592.1	1602.9	<0.0001
Population (1 000 000 people)	80.1	258.1		76.7	268.5	88.4	300.9	0.9025
Aging rate (%)	5.9	4.5		6.9	5.2	8.1	6.5	0.0923
Gross domestic product (US$ 1000/capita)	4.7	8.5		7.4	13.4	10.9	17.0	0.0654
Age-standardised education (year)	5.4	3.7		7.2	4.1	8.4	4.0	0.0017
Mean systolic blood pressure (mmHg)	128.2	3.8		129.3	3.7	129.8	3.2	0.0712
Age-standardised smoking prevalence (%)	16.2	8.7		15.9	8.0	14.3	7.2	0.3158
Age-standardised total physical activity (MET-min/week/1000)	5.3	1.6		5.7	1.8	5.8	1.7	0.2368
Mean body mass index (kg/m^2^)	23.2	1.9		24.1	2.0	24.8	2.0	0.0011
Energy supply (kcal/d/capita)	2.5	0.6		2.6	0.5	2.8	0.4	0.0107
Alcohol supply (ethanol g/d/capita)	79.2	122.3		94.1	133.6	101.8	125.2	0.4665
G3 (41 countries, in which the T2DM prevalence trends large increase)*								
Age-standardised prevalence of type 2 diabetes (per 100 000)	5824.1	1347.6		7100.4	1560.4	8203.1	1668.4	<0.0001
Population (1 000 000 people)	30.2	54.8		31.7	58.9	37.6	67.9	0.6026
Aging rate (%)	5.1	4.1		6.5	4.7	7.4	5.8	0.0497
Gross domestic product (US$1000/capita)	5.2	8.6		6.9	12.8	9.5	15.1	0.1405
Age-standardised education (year)	5.2	3.2		7.3	3.3	8.6	3.0	<0.0001
Mean systolic blood pressure (mmHg)	129.2	3.7		129.6	3.5	130.0	3.5	0.3148
Age-standardised smoking prevalence (%)	15.3	7.9		14.2	7.0	13.8	7.2	0.3703
Age-standardised total physical activity (MET-min/week/1000)	4.8	1.3		5.3	1.8	5.4	1.7	0.0936
Mean body mass index (kg/m^2^)	23.7	1.6		24.8	1.6	25.5	1.8	<0.0001
Energy supply (kcal/d/capita)	2.5	0.5		2.7	0.5	2.8	0.5	0.0063
Alcohol supply (ethanol g/d/capita)	78.2	121.8		73.4	103.5	76.6	90.0	0.9450

MET – metabolic equivalent, T2DM – type 2 diabetes mellitus, SD – standard deviation

*Based on the slopes according to the year from 1990 to 2017 for age-standardised prevalence of type 2 diabetes evaluated using a general linear model; 139 countries were classified as decrease or no change (G0), slight increase (G1), moderate increase (G2) and large increase (G3).

†The annual trends from 1990 to 2017 were tested using a generalised linear model.

**Table 2** shows the main effects and interactions for the year and the four groups on the socioeconomic, lifestyle and health indicators. The main effect of the year was significant for all variables (*P* < 0.01 for all), and only smoking prevalence in fixed effects was negatively associated, otherwise positively associated (data not shown). Among the main effects of the four groups from G0 to G3, the ageing rate, education, smoking prevalence and BMI were significant (*P* < 0.05 for all), and these factors in fixed effects were negatively associated (data not shown). The interaction between the year and the four groups was significant for variables, except energy supply (*P* < 0.01 for all).

**Table 2:** The main effects and interactions of the year and the four groups according to type 2 diabetes prevalence trends (G0 to G3)* on socioeconomic, lifestyle and health indicators

Socio-economic, life-style and health indicators	*F*-values*					
**Year**	***P*-value**	**Group^†^**	***P*-value**	**Year: Group**	***P*-value**
Aging rate (%)	116.56	<0.0001	4.11	0.0079	4.82	0.0024
Gross domestic product (US$1000/capita)	1535.06	<0.0001	1.11	0.3475	34.13	<0.0001
Age-standardised education (year)	30 796.92	<0.0001	4.14	0.0076	95.04	<0.0001
Mean systolic blood pressure (mmHg)	10.01	0.0016	0.36	0.7814	32.45	<0.0001
Age-standardised smoking prevalence (%)	1710.44	<0.0001	3.59	0.0154	49.22	<0.0001
Age-standardised total physical activity (MET-min/week/1000)	606.03	<0.0001	2.12	0.1011	19.83	<0.0001
Mean body mass index (kg/m^2^)	13 911.85	<0.0001	2.71	0.0478	66.68	<0.0001
Energy supply (kcal/d/capita)	2208.29	<0.0001	1.58	0.1980	2.08	0.1004
Alcohol supply (ethanol g/d/capita)	174.18	<0.0001	2.57	0.0566	17.68	<0.0001

MET – metabolic equivalent, SD – standard deviation

*Type III tests of fixed effect of the year, the four groups, and their interactions for each of the socioeconomic, lifestyle and health indicators estimated by F-values using a generalised linear mixed-effects model with random intercept by country and random slope by year. The dependent and independent variables were mean-centred.

†Based on the slopes according to the year from 1990 to 2017 for age-standardised prevalence of type 2 diabetes evaluated using a general linear model; 139 countries were classified as decrease or no change (G0), slight increase (G1), moderate increase (G2) and large increase (G3).

The results of the generalised linear mixed-effects model for the age-standardised prevalence of T2DM are shown in **Table 3**. The backward stepwise selection method removed the factors alcohol supply and energy supply from the model. For the age-standardised prevalence of T2DM, in the order of variables with a higher effect, education (*P* < 0.0001) and physical activity (*P* < 0.0001) were negatively associated, whereas BMI (*P* < 0.0001), SBP (*P* < 0.0001), ageing rate (*P* = 0.0004), smoking prevalence (*P* = 0.0960) and GDP (*P* = 0.0197) were positively associated. The estimated standardised effect of education was the largest at −524.5 (SE = 109.9), followed by −514.8 (SE = 95.6) for physical activity and 398.8 (SE = 45.8) for BMI.

**Table 3:** Longitudinal association between age-standardised prevalence of type 2 diabetes mellitus and socioeconomic, lifestyle and health indicators in 139 countries from 1990 to 2017

Model*	Standardised fixed effects†	SE	*P*-value
(Intercept)	6069.5	139.6	<0.0001
Aging rate (%)	165.9	46.9	0.0004
Gross domestic product (US$1000/capita)	34.0	14.6	0.0197
Age-standardised education (year)	-524.5	109.9	<0.0001
Mean systolic blood pressure (mmHg)	133.7	23.6	<0.0001
Age-standardised smoking prevalence (%)	51.1	30.7	0.0960
Age-standardised total physical activity (MET-min/week/1000)	-514.8	95.6	<0.0001
Mean body mass index (kg/m^2^)	398.8	45.8	<0.0001
Year	396.5	36.8	<0.0001

MET – metabolic equivalent, SE – standard error

*A generalised linear mixed-effects model was used with random intercept by country and random slope by year. The model was selected using the backward stepwise selection method, and energy and alcohol supply were excluded.

†Standardised independent variables (mean, 0; standard deviation, 1) were used in the analysis.

## DISCUSSION

This international comparative study demonstrated that the age-standardised prevalence of T2DM exhibited an increasing trend worldwide, but the slope varied according to the country, and education, physical activity and BMI were the most relevant in that order on the prevalence and trends in each country. Although several studies have explored the prevalence and trends of diabetes mellitus at the global level [1,7-9], there are no studies that have investigated the factors associated with them.

In this longitudinal ecological study, in the four groups divided according to the degree of increase in the age-standardised prevalence rates of T2DM, the mean prevalence in 1991 (change to 2017) were 4952.1 (279.6) for G0, 5105.7 (844.7) for G1, 4921.3 (1670.7) for G2 and 5824.1 (2379.0) for G3. Age-standardised education growth rates in 1991 (change to 2017) were 8.0 (2.4) for G0, 7.3 (2.9) for G1, 5.4 (3.0) for G2 and 5.2 (3.4) for G3. Therefore, although the growth rate of education was higher in G2 and G3 than in G0, several countries had lower levels of education. Similarly, several prospective cohort studies have reported that education level correlated inversely with the development of diabetes independently of behavioural factors such as body size, physical activity, diet, smoking and alcohol use [16-19]. However, education level was not mentioned in recent reviews of risk factors for diabetes and in the American Diabetes Association (ADA)-recommended criteria for testing for pre-diabetes or T2DM in asymptomatic adults [20,21]. This is probably because education level is not a remediable factor at the individual level in adults. It is possible that international action could be taken to close country-level education gaps and approach towards curbing the increase in global diabetes prevalence. Therefore, the promotion of policies that reduce international educational disparities may have the potential to reduce the global increase in the number of patients with T2DM.

Independent of education, physical activity and BMI were significantly associated with the age-standardised prevalence and trends of T2DM in each country as follows: in 1991, the mean physical activity levels (change to 2017) were 5.1 (0.9) for G0, 6.0 (0.2) for G1, 5.3 (0.5) for G2 and 5.2 (0.6) for G3, whereas the BMI values (change to 2017) were 24.6 (1.5) for G0, 24.3 (1.4) for G1, 23.2 (1.6) for G2 and 23.7 (1.8) for G3. These results suggest that the low levels of physical activity in G2 and G3 showed no large increase and therefore, resulted in a large increase in BMI and an increase in T2DM prevalence. These results suggest that a focus on national strategies that increase physical activity and do not increase BMI, which are recommended to improve health, may help to curb the increasing prevalence of diabetes in the world.

According to the IDF, there are regional differences in the increase in the number of people with diabetes, with higher rates of increase in Africa and South East Asia [1]. The present study also identified the four groups according to GBD super regions for the 139 targeted countries to provide information on the regions to be focused on (Table S2 in the **Online Supplementary Document**). ‘North Africa and the Middle East’ had the highest proportions of G3 at 56.2% and 81.2% when G2 was added. This was followed by ‘Southeast Asia, East Asia and Oceania’, where G3 comprised 50.0% and 83.3% when G2 was added. This was followed by ‘sub-Saharan Africa’ at 80.0% for G3 and G2. Similar to the report on the prevalence of diabetes according to region in the IDF, a regional pattern of trends in the age-standardised prevalence of T2DM was confirmed in the present study. However, because all regions have G3 countries, each country should take measures according to its own socioeconomic, lifestyle and health indicators.

Regarding the limitations of the present investigation, it was an ecological study conducted at the population level. To avoid the ecological fallacy, it should be noted that the factors strongly associated with T2DM trend in the present study were, but not individual-level, population-level factors. Therefore, we could not analyse the age-standardised prevalence of T2DM and the factors associated with its trend according to gender and race in each country. However, ecological studies have the following advantages: they control for some of the biases associated with survey research, and they allow for long-term follow-up, which is difficult to achieve in clinical trials. Therefore, this study was not hypothesis testing, but rather hypotheses, which supporting public policy, were derived from long-term, global data. We believe that the findings of this study would provide information that may not be obtained from cohort or clinical studies. Other limitations include the failure to consider differences in the period of compulsory education in each country and other factors that may affect T2DM (eg, efforts to control T2DM in each country such as health education implementation rates). Perhaps the factors used in this study (eg, education and physical activity) could be a proxy for unavailable information. Moreover, countries with a population of less than 1 million were excluded to account for the accuracy of the data.

To our knowledge, there are no studies that have investigated the factors associated with the prevalence of T2DM in global countries. The present study identified the association between the trends of T2DM prevalence and socioeconomic, lifestyle and health indicators according to country, and proposed hypotheses for discussing public health approaches to control the increase in global T2DM prevalence. The associations of education, physical activity, and BMI with T2DM prevalence and trends in each country were found to be particularly significant. We believe that the results of this study would provide a useful basis for considering policies targeting the increase in the prevalence of T2DM at both global and national levels.

## Additional material

Online Supplementary Document
