# Supplementary material for: Trends in age-standardised prevalence of type 2 diabetes mellitus according to country from 1990 to 2017 and their association with socioeconomic, lifestyle and health indicators: An ecological study
Source: J Glob Health. 2021 Jan 31;11:04005. doi: 10.7189/jogh.11.04005 (PMC7915945; doi:10.7189/jogh.11.04005)
Supplement: Online Supplementary Document [file jogh-11-04005-s001.pdf]

Table S1. The trends on age-standardised prevalence of type 2 diabetes by 139 countries with a population of more than 1 million people and four groups according to the estimated slopes.

Country	Slope*	SE	*P*-value	Group[†]
Afghanistan	61.5	11.7	<0.0001	2
Albania	29.8	27.8	<0.0001	1
Algeria	86.6	17.3	<0.0001	3
Angola	70.7	27.0	<0.0001	3
Argentina	36.8	40.0	<0.0001	1
Armenia	52.4	6.3	<0.0001	2
Australia	-0.3	-0.1	0.9050	0
Austria	63.2	18.8	<0.0001	2
Azerbaijan	61.1	10.4	<0.0001	2
Bangladesh	38.2	5.0	<0.0001	1
Belarus	-5.0	-3.6	0.0016	0
Belgium	42.1	32.4	<0.0001	1
Benin	79.2	43.5	<0.0001	3
Bolivia (Plurinational State of)	31.8	13.7	<0.0001	1
Bosnia and Herzegovina	124.7	22.0	<0.0001	3
Botswana	100.4	34.4	<0.0001	3
Brazil	-12.7	-5.7	<0.0001	0
Bulgaria	0.6	0.1	0.8980	0
Burkina Faso	45.8	26.6	<0.0001	2
Cambodia	30.1	8.6	<0.0001	1
Cameroon	67.6	18.7	<0.0001	3
Canada	-24.7	-11.5	<0.0001	0
Central African Republic	67.1	35.6	<0.0001	3
Chad	60.2	46.4	<0.0001	2
Chile	38.0	17.7	<0.0001	1

China	45.6	6.2	<0.0001	2
Colombia	-26.6	-5.6	<0.0001	0
Congo	57.0	32.2	<0.0001	2
Costa Rica	10.3	10.8	<0.0001	1
Cote d'Ivoire	62.8	19.1	<0.0001	2
Croatia	41.9	18.9	<0.0001	1
Cuba	-9.2	-2.2	0.0387	0
Cyprus	-4.9	-0.6	0.5300	0
Czech Republic	58.0	7.9	<0.0001	2
Denmark	104.7	22.4	<0.0001	3
Dominican Republic	49.4	21.2	<0.0001	2
Ecuador	42.6	33.4	<0.0001	2
Egypt	114.8	38.1	<0.0001	3
El Salvador	61.5	26.8	<0.0001	2
Estonia	43.7	7.7	<0.0001	2
Ethiopia	-36.8	-86.7	<0.0001	0
Finland	82.7	38.3	<0.0001	3
France	45.5	72.1	<0.0001	2
Gabon	86.3	33.4	<0.0001	3
Georgia	111.3	48.5	<0.0001	3
Germany	32.7	10.4	<0.0001	1
Ghana	87.7	51.3	<0.0001	3
Greece	62.6	18.3	<0.0001	2
Guatemala	120.4	20.9	<0.0001	3
Guinea	61.3	22.8	<0.0001	2
Guinea-Bissau	68.7	31.5	<0.0001	3
Haiti	-0.3	-0.3	0.8010	0
Honduras	36.0	19.6	<0.0001	1

Hungary	32.5	9.7	<0.0001	1
India	50.0	10.0	<0.0001	2
Indonesia	83.7	7.6	<0.0001	3
Iran (Islamic Republic of)	92.8	14.1	<0.0001	3
Iraq	-11.2	-1.0	0.3360	0
Ireland	59.8	22.6	<0.0001	2
Israel	37.9	6.0	<0.0001	1
Italy	26.0	7.4	<0.0001	1
Jamaica	62.8	14.0	<0.0001	2
Japan	0.7	0.2	0.8810	0
Jordan	60.0	21.8	<0.0001	2
Kazakhstan	2.1	1.1	0.2820	0
Kenya	45.0	30.1	<0.0001	2
Kuwait	30.2	4.1	0.0004	1
Kyrgyzstan	0.2	0.1	0.9070	0
Lao People's Democratic Republic	79.3	38.9	<0.0001	3
Latvia	52.0	20.6	<0.0001	2
Lebanon	58.5	24.8	<0.0001	2
Lesotho	123.1	28.8	<0.0001	3
Liberia	67.5	51.9	<0.0001	3
Lithuania	31.6	15.2	<0.0001	1
Madagascar	37.3	57.2	<0.0001	1
Malawi	37.3	18.1	<0.0001	1
Malaysia	49.1	5.3	<0.0001	2
Mali	51.3	154.7	<0.0001	2
Mauritania	64.1	104.8	<0.0001	3
Mauritius	272.0	26.4	<0.0001	3
Mexico	31.9	4.6	0.0001	1

Mongolia	26.9	17.4	<0.0001	1
Morocco	89.6	84.3	<0.0001	3
Mozambique	50.9	37.5	<0.0001	2
Myanmar	56.2	12.6	<0.0001	2
Namibia	51.8	17.6	<0.0001	2
Nepal	29.1	15.5	<0.0001	1
Netherlands	52.1	29.4	<0.0001	2
New Zealand	-18.0	-2.9	0.0074	0
Nicaragua	-0.4	-0.2	0.8090	0
Niger	49.3	46.4	<0.0001	2
Nigeria	29.8	19.1	<0.0001	1
Norway	28.1	10.0	<0.0001	1
Oman	104.1	23.8	<0.0001	3
Pakistan	105.7	22.2	<0.0001	3
Panama	28.0	30.2	<0.0001	1
Paraguay	69.6	14.0	<0.0001	3
Peru	29.5	12.5	<0.0001	1
Philippines	137.2	33.9	<0.0001	3
Poland	33.3	13.0	<0.0001	1
Portugal	69.7	31.4	<0.0001	3
Romania	38.1	41.1	<0.0001	1
Russian Federation	3.1	1.5	0.1350	0
Rwanda	-7.6	-3.3	0.0028	0
Saudi Arabia	104.8	15.4	<0.0001	3
Senegal	74.3	44.2	<0.0001	3
Serbia	14.3	3.9	0.0028	1
Sierra Leone	61.2	30.8	<0.0001	2
Slovakia	20.1	22.0	<0.0001	1

Slovenia	8.7	2.5	0.0197	1
South Africa	92.3	19.0	<0.0001	3
Republic of Korea	23.4	6.5	<0.0001	1
Spain	22.7	5.3	<0.0001	1
Sri Lanka	158.7	39.1	<0.0001	3
Sudan	63.7	16.7	<0.0001	3
Sweden	57.8	145.0	<0.0001	2
Switzerland	34.5	18.4	<0.0001	1
Tajikistan	63.7	23.1	<0.0001	3
United Republic of Tanzania	44.0	36.3	<0.0001	2
Thailand	15.2	2.5	0.0174	1
Gambia	61.4	72.9	<0.0001	2
Timor-Leste	88.6	14.5	<0.0001	3
Togo	52.7	62.2	<0.0001	2
Trinidad and Tobago	8.8	2.3	0.0290	1
Tunisia	70.4	54.6	<0.0001	3
Turkey	22.2	7.5	<0.0001	1
Turkmenistan	61.0	24.4	<0.0001	2
Uganda	33.2	15.9	<0.0001	1
Ukraine	2.1	0.9	0.3830	0
United Arab Emirates	112.8	15.9	<0.0001	3
United Kingdom of Great Britain and Northern Ireland	88.4	35.9	<0.0001	3
United States of America	111.9	13.5	<0.0001	3
Uruguay	-11.8	-10.3	<0.0001	0
Uzbekistan	85.5	20.5	<0.0001	3
Venezuela (Bolivarian Republic of)	9.2	2.3	0.0303	1
Viet Nam	60.6	30.3	<0.0001	2

Yemen	42.5	11.3	<0.0001	2
Zambia	25.0	15.3	<0.0001	1
Zimbabwe	129.3	29.4	<0.0001	3

SE - standard error

* The slopes on age-standardised prevalence of type 2 diabetes were estimated using a general linear model.

[†] Countries were divided into four groups according to the estimated slope: 0, decrease or no change; 1, slight increase (the lowest tertile at the significant positive slope); 2, moderate increase (the middle tertile at the significant positive slope); and 3, large increase (the highest tertile at the significant positive slope).

Table S2. Number and percentage of countries belonging to each of the four groups according to age-standardised prevalence of type 2 diabetes by GBD Super Regions.

GBD Super Regions	Group*, n (%)			
	G0	G1	G2	G3
Central Europe, Eastern Europe, and Central Asia	6 (23.1)	10 (38.5)	6 (23.1)	4 (15.4)
High‐income	6 (22.2)	10 (37.1)	6 (22.2)	5 (18.5)
Latin America and Caribbean	5 (26.3)	8 (42.1)	4 (21.1)	2 (10.5)
North Africa and Middle East	1 (6.2)	2 (12.5)	4 (25.0)	9 (56.2)
South Asia	0 (0.0)	2 (50.0)	1 (25.0)	1 (25.0)
Southeast Asia, East Asia, and Oceania	0 (0.0)	2 (16.7)	4 (33.3)	6 (50.0)
Sub‐Saharan Africa	2 (5.7)	5 (14.3)	14 (40.0)	14 (40.0)

GBD - Global Burden of Diseases

*Based on the slopes according to the year from 1990 to 2017 for age-standardised prevalence of type 2 diabetes evaluated using a general linear model; 139 countries were classified as decrease or no change (G0), slight increase (G1), moderate increase (G2) and large increase (G3).
